# Interstitial Lung Disease and Progressive Pulmonary Fibrosis: a World Trade Center Cohort 20-Year Longitudinal Study

**DOI:** 10.1007/s00408-024-00697-z

**Published:** 2024-05-07

**Authors:** Krystal L. Cleven, Rachel Zeig-Owens, Alexandra K. Mueller, Brandon Vaeth, Charles B. Hall, Jaeun Choi, David G. Goldfarb, David E. Schecter, Michael D. Weiden, Anna Nolan, Steve H. Salzman, Nadia Jaber, Hillel W. Cohen, David J. Prezant

**Affiliations:** 1 Department of Medicine, Montefiore Medical Center, Bronx, NY USA; 2 Bureau of Health Services, Fire Department of the City of New York, 9 Metrotech Center, Brooklyn, NY 11201 USA; 3 Department of Epidemiology and Population Health, Albert Einstein College of Medicine, Bronx, NY USA; 4 New York University Grossman School of Medicine, New York, NY USA

**Keywords:** Interstitial lung disease, Pulmonary fibrosis, World trade center, Firefighters, Emergency Medical Service Providers, Progressive pulmonary fibrosis

## Abstract

**Purpose:**

World Trade Center (WTC) exposure is associated with obstructive airway diseases and sarcoidosis. There is limited research regarding the incidence and progression of non-sarcoidosis interstitial lung diseases (ILD) after WTC-exposure. ILD encompasses parenchymal diseases which may lead to progressive pulmonary fibrosis (PPF). We used the Fire Department of the City of New York’s (FDNY’s) WTC Health Program cohort to estimate ILD incidence and progression.

**Methods:**

This longitudinal study included 14,525 responders without ILD prior to 9/11/2001. ILD incidence and prevalence were estimated and standardized to the US 2014 population. Poisson regression modeled risk factors, including WTC-exposure and forced vital capacity (FVC), associated with ILD. Follow-up time ended at the earliest of incident diagnosis, end of study period/case ascertainment, transplant or death.

**Results:**

ILD developed in 80/14,525 FDNY WTC responders. Age, smoking, and gastroesophageal reflux disease (GERD) prior to diagnosis were associated with incident ILD, though FVC was not. PPF developed in 40/80 ILD cases. Among the 80 cases, the average follow-up time after ILD diagnosis was 8.5 years with the majority of deaths occurring among those with PPF (PPF: *n* = 13; ILD without PPF: *n* = 6).

**Conclusions:**

The prevalence of post-9/11 ILD was more than two-fold greater than the general population. An exposure-response gradient could not be demonstrated. Half the ILD cases developed PPF, higher than previously reported. Age, smoking, and GERD were risk factors for ILD and PPF, while lung function was not. This may indicate that lung function measured after respirable exposures would not identify those at risk for ILD or PPF.

**Supplementary Information:**

The online version contains supplementary material available at 10.1007/s00408-024-00697-z.

## Introduction

The World Trade Center (WTC) disaster exposed over 15,000 Fire Department of the City of New York (FDNY) responders (firefighters and emergency medical services (EMS) providers) to hazardous WTC dust/gases [1–3]. WTC-exposure has been found to be associated with obstructive airway diseases and sarcoidosis [4, 5]. However, there is limited research regarding the incidence and progression of non-sarcoidosis interstitial lung diseases (ILD) after WTC-exposure. Case reports have described ILD findings on pathologic examination after WTC-exposure, and mineralogic analyses identified the presence of metals, silica, aluminum silicates, carbon nanotubes, chrysotile asbestos, and calcium phosphate or sulfate [6–8], many of which are known to be associated with ILD [9–11]. Murine studies indicate that histopathologic changes, such as an increase in collagen in cardiopulmonary tissue occur after WTC particulate exposure [12, 13]. To date, there has only been one WTC-cohort study reporting an increase in pulmonary fibrosis, based solely on self-reports, without medical record confirmation, or information on radiographic presentation, lung function, or progression [14].

ILD includes a large group of disorders with varying degrees of diffuse inflammation and fibrosis, resulting in pulmonary restriction, impaired gas exchange, poor quality of life, and shortened lifespan [15, 16]. While idiopathic pulmonary fibrosis (IPF) has been associated with various exposures including drug and radiation treatment [17–21], the ATS/ERS/JRS/ALAT 2022 Clinical Practice Guideline does not recommend the use of the term IPF when the cause is related to environmental/occupational exposures [22]. Using a US healthcare claims database of over 37 million patients, with age- and sex-standardized to the US 2014 population, the prevalence of ILD was 118/100,000 persons and of PPF was 70/100,000 persons [23]. Physician surveys estimate that as many as one-third of ILD patients, after excluding those with IPF, may develop progressive pulmonary fibrosis (PPF) [24].

The FDNY-WTC cohort includes active and retired FDNY employees. It is well-designed to identify the occurrence and severity of post-9/11/2001 (9/11) ILD as medical records prior to 9/11 are available, has over 20 years of follow-up (including annual medical evaluations with spirometry, and chest radiographs every 2–3 years), and ILD is an exclusion from hire. Full pulmonary function tests (PFTs) and chest computerized tomography (CT) are obtained as clinically indicated. Using this extensive database, the goals of this longitudinal study was to estimate the incidence and prevalence of ILD following WTC-exposure; describe the clinical characteristics of ILD; and identify risk factors associated with ILD or PPF. We hypothesized that lung function measured prior to diagnosis would be a risk factor predicting the development of ILD and/or PPF.

## Methods

### Source Population

The source population included firefighters and EMS providers present at the WTC-disaster site for at least one day between 9/11/2001 and 7/24/2002 who provided written informed consent. Exclusion criteria included participants with any of the following: ILD diagnosed pre-9/11, sarcoidosis, or drug- or radiation-induced ILD. The final study population was 14,525 participants. The Albert Einstein College of Medicine/Montefiore Medical Center Institutional Review Board approved the study.

### Interstitial Lung Disease Case Definition

Due to post-9/11 symptoms, decline in lung function (annual spirometry), and/or abnormal chest radiographs, 6,655 workers had ≥ 1 CT scans and 4,282 had ≥ 2 CTs. All CTs conducted between 9/11/2001 and 3/31/2023 (end of follow-up) were interpreted by board-certified radiologists, reviewed by our senior board-certified pulmonologist, and entered into the FDNY-WTC database. All CT results with the keywords of “fibrosis, UIP, UIP with honeycombing, NSIP, or reticulation” were extracted and reviewed to confirm they met CT criteria for ILD (honeycombing, traction bronchiectasis, or reticular infiltrates with/without diffuse ground glass opacities) with required confirmation on more than one CT scan [15, 16]. As we were interested in defining risk for progression, participants were included even if their lung function was normal at the time of diagnosis by CT. This case definition excludes isolated interstitial lung abnormalities by not including cases with unilateral or focal CT findings or ground glass opacities without bilateral reticular findings and by requiring confirmation on repeat CT or biopsy. To minimize the possibility of including ILD cases from Coronavirus Disease 2019 (COVID-19), we excluded cases diagnosed after 3/1/2020 (*n* = 26). The FDNY-WTC database (medical records and claims data) was also searched for participants with any ICD-10 diagnosis code indicating ILD or progression, but no additional cases were identified. After applying these criteria, we identified 80 post-9/11 ILD participants.

### Spirometry and PFTs

Spirometry was obtained during participants’ medical monitoring exams every 12–18 months. Spirometry was performed as described elsewhere [25] and required a quality grade of A, B, or C to be included in analyses. The main measure of interest was forced vital capacity (FVC) in liters and percent predicted (FVC%), using NHANES III reference equations [26]. If a participant had more than one spirometry with reliable FVC measurements between 1/1/1998 and 9/10/2001, the one closest to 9/10/2001 was used. PFTs (FVC, total lung capacity [TLC], and diffusing capacity of the lungs for carbon monoxide [DLCO]) were obtained upon referral for diagnosis/treatment. Post-transplant PFTs, including spirometry, were excluded. Values ≥ 80% predicted were considered “normal.”

### Progressive Pulmonary Fibrosis Case Definition

Each case was reviewed to identify participants who met the American Thoracic Society’s criteria for PPF which required two of the following: (1) physiological evidence of disease progression (PFT with either ≥ 5% FVC decline or ≥ 10% DLCO decline over 1 year), (2) radiological evidence of disease progression (CT findings of honeycombing, worsening traction bronchiectasis, or worsening reticular infiltrates), and (3) worsening respiratory symptoms [22, 27]. For worsening respiratory symptoms, because all participants reported dyspnea and many reported some degree of progression, we confirmed progression severity by including only those participants prescribed home oxygen (oxygen saturation < 88%), anti-fibrotic medications, or having received a lung transplant. PPF diagnosis date was identified as the first date one met the above criteria.

### Covariate Data

Demographic/clinical characteristics including sex, race, birth date, death date, use of home oxygen, medication history, transplant date, and gastroesophageal reflux disease (GERD) diagnosis prior to an ILD diagnosis were obtained from the FDNY-WTC database. WTC-exposure was self-reported at participants’ first post-9/11 health questionnaire and defined based on initial arrival and/or duration at the WTC-site [28]. The first reported smoking status (former, current, or never) was used. Cause of death was obtained from the National Death Index through 2021; after 2021, FDNY records were used for death date.

### Statistics

We used proportions and means(± SD) to describe characteristics of those without and with ILD, the latter stratified by PPF. Since ILD results in symptoms and physiological abnormalities that lead to CT assessment, prevalence and incidence was estimated using the entire WTC cohort and not restricted to those who received CTs. Prevalence was estimated for all alive participants at the end of case ascertainment (3/1/2020). Incidence was estimated from 9/11 until 3/1/2020. Person time accrual for the incidence rate began on 9/12/2001 and ended at the earliest date of ILD diagnosis, death, or 3/1/2020. PPF prevalence was estimated for all living participants at the end of follow-up (3/31/2023) as one could have progressed after the end of case ascertainment. PPF incidence was estimated from 9/12/2001 to the earliest of date of PPF, death, or 3/31/2023. For comparison with prior literature [23], rates were age- and sex-standardized to the US Census Bureau 2014 National Population Projections.

Change in absolute FVC was estimated for all participants with post-9/11 spirometry measurements using mixed linear effects models. *Post-9/11 FVC change* was estimated using follow-up from 9/11/2002 to the earliest date of ILD diagnosis (when applicable), death, or end of follow-up (3/31/2023). Follow-up for these analyses began on 9/11/2002 as the spirometry instrument was changed, as described elsewhere [25]; similarly, individual *post-ILD-diagnosis FVC change* from ILD diagnosis date to the earliest date of lung transplant, death or end of follow-up (3/31/2023) was estimated for ILD participants with post-ILD spirometry. Models included age on 9/11, height, weight, sex (male as the reference), and race (White as the reference) as fixed effects. Consistent with our prior work, we estimated within-subject variability using intercept-only linear mixed model variance components [25]. To visually present longitudinal differences in FVC% by PPF status, we graphed mean FVC% by year following ILD diagnosis.

### Predicting ILD Occurrence

Predictors of ILD were evaluated as relative rates (RRs) estimated by Poisson regression. Multivariable Poisson regression models included lung function, age on 9/11, smoking status, WTC-exposure (arrival time, duration, and two binary composite variables - those who arrived at the site any time on 9/11 and/or worked longer than 3 months/those who arrived on 9/12 or later and/or worked fewer than 3 months), and GERD prior to ILD diagnosis. Each Poisson model was run separately using different FVC measures– in liters and as % predicted; last pre-9/11; first post-9/11; and trans-9/11 FVC (first post-9/11 minus last pre-9/11). Nine separate models were fitted for each WTC-exposure and lung function combination. Additionally, because WTC-exposure may cause GERD, we fit models with and without GERD [29, 30]. Person-time for the ILD models began on first FVC measurement and ended at the earliest date of ILD diagnosis, death, or 3/1/2020 (case ascertainment). Person-time was included as an offset in the models.

### Predicting PPF Occurrence

Multivariable Poisson regression models included age on 9/11, smoking status, WTC-exposure, and GERD prior to ILD diagnosis. FVC was not included as a covariate in these models as it is part of the ATS definition for PPF. Person-time for the PPF models began on the date of ILD diagnosis and ended at the earliest date of PPF, death, or 3/31/2023 as a participant could have progressed after the end of case ascertainment. Person-time was included as an offset in the models. Because FVC (change over time) is in the definition of PPF but is also a variable of interest as a potential predictor of PPF, we conducted a sensitivity analysis by repeating the above analyses defining ILD participants solely by CT criteria for fibrosis (presence or absence of honeycombing and/or traction bronchiectasis).

### Including Sarcoidosis Participants

Because the largest prior study estimating ILD incidence/prevalence rates included sarcoidosis patients [23], two additional sensitivity analyses were performed. In total, 99 participants with biopsy-proven sarcoidosis and any lung involvement diagnosed post-9/11 were added to the population (*n* = 14,624). The first analysis attempted to duplicate the prior study [23] by adding the 99 participants with sarcoidosis and any type of lung involvement to the original 80 cases, for a total of 179 cases. The second did what the prior study could not, adding only those meeting criteria for ILD (*n* = 5), for a total of 85 cases. All analyses were then run with these populations. Analyses were performed using SAS version 9.4 (SAS Institute, Cary, NC, USA, http://www.sas.com).

## Results

Out of 14,525 FDNY-WTC-responders, 80 participants met CT criteria for ILD (Table 1). In March 2020, the crude age-specific post-9/11 prevalence rates of ILD increased with age (e.g., 50–59 years: 95.4/100,000 persons and 60–69 years: 630.9/100,000 persons). The age- and sex-standardized prevalence rate was 252.5/100,000 persons (Table 2). The age- and sex-standardized incidence rate for ILD was 37.0/100,000 person-years. The age- and sex-standardized prevalence rate of PPF was 80.9/100,000 persons and the age- and sex-standardized incidence rate for PPF was 11.5/100,000 person-years (Table 2). These incidence rates for ILD and PPF increased with age up to age 70.

**Table 1:** Demographic and clinical characteristics of WTC-exposed FDNY Cohort by ILD and PPF status

	ILD Status		PPF Status Among ILD cases	
	**No ILD**	**All ILD**	**PPF**	**Without PPF**
Total	14,445	80	40	40
Male Sex	13,999 (96.9%)	79 (98.8%)	40 (100%)	39 (97.5%)
*Race*				
White	12,583 (87.1%)	76 (95%)	40 (100%)	36 (90%)
Black	777 (5.4%)	2 (2.5%)	0	2 (5%)
Hispanic	948 (6.6%)	2 (2.5%)	0	2 (5%)
Other	137 (0.9%)	0	0	0
Mean age on 9/11 (SD)	40 (9.5)	53 (8.2)	53 (7.3)	52 (9.2)
Mean age at ILD Diagnosis (SD)		66 (9.0)	66 (8.4)	66 (9.7)
Mean years after 9/11 until diagnosis (SD)		13.0 (4.3)	12.5 (4.1)	13.4 (4.5)
*Smoker*				
Ever	5549 (38.7%)	56 (70%)	30 (75.0%)	26 (65.0%)
Never	8804 (61.3%)	24 (30%)	10 (25.0%)	14 (35.0%)
*Employment Class*				
EMS	1998 (13.8%)	4 (5.0%)	2 (5.0%)	2 (5.0%)
Firefighter	12,437 (86.1%)	76 (95.0%)	38 (95.0%)	38 (95.0%)
GERD	6811 (47.2%)	62 (77.5%)	29 (72.5%)	33 (82.5%)
*Arrival Time at WTC site*				
Morning of 9/11	2235 (15.5%)	9 (11.3%)	2 (5.0%)	7 (17.5%)
Afternoon of 9/11	6568 (45.5%)	33 (41.3%)	20 (50.0%)	13 (32.5%)
9/12	2609 (18.1%)	14 (17.5%)	7 (17.5%)	7 (17.5%)
9/13 − 9/24	2481 (17.2%)	20 (25%)	10 (25.0%)	10 (25.0%)
After 9/24	441 (3.1%)	3 (3.8%)	1 (2.5%)	2 (5.0%)
Unknown	111 (0.8%)	1 (1.3%)	0 (0)	1 (2.5%)
Duration at WTC site ≥ 3 months	7055 (48.8%)	23 (28.8%)	11 (27.5%)	12 (30.0%)
Duration at WTC site < 3 months	7390 (51.2%)	57 (71.3%)	29 (72.5%)	28 (70.0%)
*Spirometry Measures*				
Pre-9/11 FVC % Pred mean (SD)^a^	98.4 (12.8)	94.7 (12.9)	95.4 (13.6)	94.1 (12.5)
First post-9/11 FVC % Pred mean (SD)	94.1 (12.5)	88.9 (14.1)	90.8 (16.2)	87.0 (11.7)
Most Recent FVC % Pred mean (SD)^b^	91.9 (65.6)	80.3 (20.2)	78.1 (23.6)	82.5 (16.2)
*Mean Change in FVC*				
Post-9/11 (95% CI)	-30.8 mL/Year (-31.7, -29.8)	-35.9 mL/Year (-43.1, -28.7)	N/A^g^	N/A^g^
Post-ILD-diagnosis (95% CI)		-79.2 mL/Year (-94.0, -64.3)	N/A^g^	N/A^g^
*Lung Function*				
FVC Decline ≥ 5%^c^			39 (100%)	38 (95.0%)
DLCO Decline ≥ 10%^d^			18 (54.6%)	13 (41.9%)
*Vital Status*				
Alive	13,407 (92.8%)	61 (76.3%)	27 (67.5%)	34 (85.0%)
Deceased^e^	1038 (7.2%)	19 (23.8%)	13 (32.5%)	6 (15.0%)
Respiratory related death	99 (10.6%)	6 (42.8%)	4 (40.0%)	2 (50%)
Other causes of death	839 (89.4%)	8 (57.1%)	6 (60.0%)	2 (50%)
Total person time (years)^f^	254795.7	1037.6	615.8	837.2
Mean person time (years)^f^	17.6	13.0	15.4	20.9
On home oxygen		15 (18.8%)	15 (37.5%)	0 (0)
Anti-fibrotic medication		17 (21.3%)	17 (42.5%)	0 (0)
Received lung transplant		7 (8.8%)	7 (17.5%)	0

ILD, interstitial lung disease; PPF, progressive pulmonary fibrosis; SD, standard deviation; EMS, Emergency Medical Services; GERD, gastroesophageal reflux disease; WTC, World Trade Center; FVC, forced vital capacity; FVC % Pred, forced vital capacity percent predicted; DLCO, diffusing capacity of the lungs for carbon monoxide

^a^Data available for 11,228 non-ILD, 21 PPF and 26 people in non-PPF

^b^Spirometry measurement closest to the end of follow-up

^c^Data available for 39 PPF and 40 non-PPF

^d^Data available for 33 PPF and 31 non-PPF

^e^Cause of death data available for 938 non-ILD, 9 PPF and 4 non-PPF

^f^Non-ILD and ILD person time calculated to 3/1/2020 (end of case ascertainment); PPF and Non-PPF-ILD person time calculated to 3/31/2023 (end of study)

^g^Too few cases to calculate

**Table 2:** Post-9/11 ILD and PPF Prevalence and Incidence Rates among WTC-exposed FDNY Cohort

	**ILD**		**PPF**	
Crude Age-Specific	2020 Prevalence Rates per 100,000 persons (95% CI)^a^	Incidence Rates per 100,000 person-years (95% CI)^b^	2023 Prevalence Rates per 100,000 persons (95% CI)^c^	Incidence Rates per 100,000 person-years (95% CI)^d^
40 to 49 years	0	3.4 (1.1, 10.4)	0	0
50 to 59 years	95.4 (39.7, 229.2)	22.7 (14.3, 36.0)	20.8 (2.9, 148.0)	4.2 (1.6, 11.2)
60 to 69 years	630.9 (435.1, 914.8)	89.0 (62.2, 127.3)	97.1 (40.4, 233.3)	39.0 (24.9, 61.2)
70 to 79 years	2247.2 (1,523.4, 3314.7)	306.7 (208.7, 450.8)	958.6 (585.9, 1568.4)	117.1 (70.6, 194.3)
80 to 89 years	3,750.1 (1,927.6, 7294.9)	296.3 (95.4, 920.4)	1243.8 (514.9, 3004.4)	102.4 (25.6, 409.9)
Age- and sex-standardized	252.5 (197.1, 307.8)	37.0 (24.4, 38.1)	80.9 (55.8, 105.9)	11.5 (7.3, 15.6)

Rates were standardized to the US Census Bureau 2014 National Population Projections

ILD, interstitial lung disease; PPF, progressive pulmonary fibrosis; WTC, World Trade Center; FDNY, Fire Department of the City of New York

^a^ILD prevalence was estimated for all alive participants at the end of case ascertainment (3/1/2020)

^b^ILD incidence was estimated from 9/11 until 3/1/2020. Person time accrual for the incidence rate began on 9/12/2001 and ended at the earliest date of ILD diagnosis, death, or 3/1/2020 (case ascertainment)

^c^PPF prevalence was estimated for all alive participants at the end of the study (3/31/2023) as one could have progressed after the end of case ascertainment

^d^PPF incidence was estimated from 9/12/2001 to the earliest of date of PPF, death, or 3/31/2023

The demographic and clinical characteristics of the cohort by ILD status are shown in Table 1. ILD diagnoses occurred on average 13 years (SD = 4.3) after 9/11, at an average age of 66 years (SD = 9.0). Most ILD participants were male (98.8%) firefighters (95%), consistent with the sex and employment distribution of our entire FDNY-WTC cohort. The mean age on 9/11 of those who developed ILD was older than those without (53 vs. 40 years old). A greater proportion of ILD participants were ever-smokers (70.0% vs. 38.7%). Just over half of those diagnosed with ILD initially arrived at the WTC-site on 9/11 (52.6%) when exposure was most intense. Mean post-9/11 change in FVC was a decline of 30.8mL/year in those who never developed ILD during the follow-up period. For those who developed ILD, the mean change in FVC was a decline of 35.9mL/year pre-diagnosis and 79.2 ml/year post-diagnosis. Multivariable Poisson models showed older age on 9/11, smoking status, and a GERD diagnosis prior to ILD diagnosis were risk factors for ILD (Table 3). Neither WTC-exposure nor FVC predicted a significant increased risk for ILD (data from analyses with alternative WTC-exposure and FVC definitions not shown).

**Table 3:** Multivariable Poisson model evaluating risk factors for post-9/11 all ILD and PPF only among WTC-exposed FDNY Cohort

	All ILD (both PPF and ILD without PPF) vs non-ILD		PPF only vs ILD without PPF	
	Relative Rate (95% CI)	Relative Rate (95% CI)	Relative Rate (95% CI)	Relative Rate (95% CI)
		Without GERD		Without GERD
First Post-9/11 FVC % Predicted	0.99 (0.98–1.01)	0.99 (0.98–1.01)	N/A	N/A
Arrived on Day 1 vs. later	1.14 (0.69–1.89)	1.21 (0.73–1.98)	1.13 (0.59–2.19)	1.13 (0.59–2.18)
Age on 9/11				
30–39 vs. 40–49	0.12 (0.04–0.40)	0.11 (0.03–0.38)	0.48 (0.06–3.73)	0.49 (0.06–3.73)
50–59 vs. 40–49	2.89 (1.62–5.15)	2.70 (1.51–4.81)	0.98 (0.45–2.14)	0.99 (0.46–2.12)
60–69 vs. 40–49	10.76 (5.51–21.01)	9.06 (4.69–17.49)	1.31 (0.54–3.20)	1.31(0.53–3.20)
GERD	4.88 (2.61–9.12)	N/A	0.98 (0.46–2.04)	N/A
Former vs. never smoking	2.47 (1.41–4.32)	2.67 (1.54–4.65)	1.39 (0.64–3.00)	1.38 (0.64–2.97)
Current vs. never smoking	2.92 (1.48–5.78)	2.70 (1.37–5.33)	1.52 (0.59–3.91)	1.52 (0.59–3.91)

ILD, interstitial lung disease; FVC, forced vital capacity; GERD, gastroesophageal reflux disease; PPF, progressive pulmonary fibrosis; WTC, World Trade Center; FDNY, Fire Department of the City of New York

On CT, many ILD participants had honeycombing (23.8%) or traction bronchiectasis (40.0%), with 13 (16.3%) having both. Reticular infiltrates were present in 98.8% and subpleural or basilar predominance in 81.3% (Table 4). On spirometry, ILD participants FVC% pre-9/11 and shortly after 9/11 were on average > 85% predicted. 66 ILD participants had full PFTs post-ILD diagnosis (Table 4). On average, lung volumes (FVC and TLC) were ≥ 80% predicted and DLCO was mildly decreased at 63% (SD = 25.1).

**Table 4:** Most Recent Post-ILD Diagnosis Pulmonary Function Tests Results and Radiographic Features

	**All ILD**	**PPF**	**ILD without PPF**
*FVC (Mean (SD))* ^a^			
Liters	3.55 (1.1)	3.36 (1.1)	3.74 (1.0)
FVC%	88.6 (24.2)	86.27 (28.6)	91.28 (18.2)
*FEV1 (Mean (SD))* ^a^			
Liters	2.67 (0.7)	2.57 (0.8)	2.79 (0.7)
FEV1%	88.18 (23.6)	87.1 (26.4)	89.32 (20.4)
*TLC (Mean (SD))* ^a^			
Liters	5.4 (1.4)	4.91 (1.3)	5.89 (1.3)
TLC%	80.1 (20.3)	75.23 (20.8)	85.19(18.7)
*DLCO (Mean (SD))* ^a^			
ml/mmHg/min	15.8 (7.4)	13.82 (5.8)	17.92 (8.3)
DLCO%	62.8 (25.1)	55.44 (20.9)	70.5(27.0)
*Radiographic Features (n (%))* ^b, c^			
Honeycombing	19 (23.8%)	19 (47.5%)	0 (0%)
Traction bronchiectasis	32 (40.0%)	24 (60.0%)	8 (20.0%)
Reticular infiltrates	79 (98.8%)	39 (97.5%)	40 (100%)
Subpleural and basilar predominance	65 (81.3%)	36 (90.0%)	29 (72.5%)

ILD, interstitial lung disease; PPF, progressive pulmonary fibrosis; FVC, forced vital capacity; L, liters; SD, standard deviation; FVC%, forced vital capacity percent predicted; FEV1, forced expiratory volume in one second; FEV1%, forced expiratory volume in one second percent predicted; TLC, total lung capacity; TLC%, total lung capacity percent predicted; DLCO, diffusing capacity of the lungs for carbon monoxide; DLCO%, diffusing capacity of the lungs for carbon monoxide percent predicted

^a^All ILD *n* = 66; PPF *n* = 34; ILD without PPF *n* = 32

^b^All ILD *n* = 80; PPF *n* = 40; ILD without PPF *n* = 40

^c^Some participants may have overlapping features

PPF developed in 40 (50.0% (95% CI: 38.6-61.4%)) ILD participants. The demographic and clinical characteristics of the cohort by PPF status are shown in Table 1. Multivariable Poisson models showed no significant risk factors for predicting PPF (Table 3). On CT, 19/40 (47.5%) had honeycombing, 24 (60.0%) had traction bronchiectasis, with 13 (32.5%) having both (Table 4). ILD with and without PPF were a similar age on 9/11 and at time of ILD diagnosis. On spirometry, PPF participants had, on average, normal FVC% pre-9/11 and shortly after 9/11 (Table 1). Change in FVC over time, regardless of when measured, was also similar between ILD participants with and without IPF. In PPF participants with full PFTs, on average, FVC, TLC, and DLCO were lower (expressed in liters or as % predicted), respectively, when compared with ILD without PPF participants (Table 4).

**Fig. 1:**
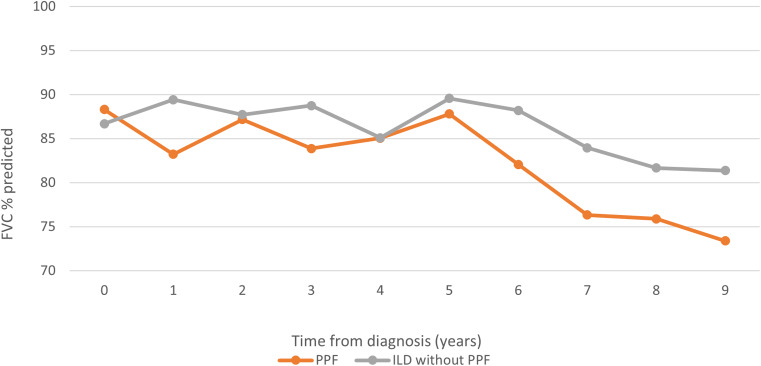
Mean FVC % predicted per year from time of post-9/11 ILD diagnosis by PPF status. Due to small numbers after year 9, follow-up after diagnosis was restricted to 9 years. Abbreviations: FVC, forced vital capacity; ILD, interstitial lung disease; PPF, progressive pulmonary fibrosis

The average FVC% post-diagnosis was consistently < 90% in persons with ILD. Those without ILD remained on average > 90% FVC% during all 21 years of follow-up (data not shown). Additionally, those with PPF, starting ~ five years after ILD diagnosis, experienced greater decline in FVC% than ILD without PPF participants (Fig. 1). The average follow-up time post-ILD diagnosis was 8.5 years with the majority of deaths in this group among those with PPF (PPF: *N* = 13; ILD without PPF: *N* = 6).

In sensitivity analyses defining ILD participants solely by CT findings (honeycombing and/or traction bronchiectasis), 38 (47.5%) had honeycombing and/or traction bronchiectasis (Online Resource 1). Sensitivity analyses findings were similar to PPF vs. ILD without PPF (data not shown).

In sensitivity analyses including sarcoidosis, of 14,624 participants, 99 had sarcoidosis with any type of lung involvement, of whom 5 had ILD, of whom 3 had PPF (Online Resources 2&3). The age and sex-standardized prevalence rate (per 100,000 persons) of sarcoidosis with ILD was 311.2 and increased to 507.2 when including sarcoidosis with any lung involvement (Online Resources 4&5). The age and sex-standardized incidence rate (per 100,000 person-years) of sarcoidosis with ILD was 37.2 and increased to 69.6 when including sarcoidosis with any lung involvement (Online Resources 4&5). Incidence and prevalence rates for PPF (Online Resources 4&5) and findings from all prediction models were similar to primary analyses (data not shown).

## Discussion

This study is the first to describe post-9/11 ILD incidence, prevalence, demographics, and radiographic features in a closed occupational cohort followed longitudinally over 21 years after an intense occupational/environmental exposure (WTC). We found an age- and sex-standardized post-9/11 prevalence rate of 252.5/100,000 persons, more than two-fold greater than most reports [23, 31], despite excluding sarcoid-related ILD from our primary analyses. Most importantly, half of the ILD cases developed PPF, higher than some previous reports [24].

Incidence and prevalence data for ILD and PPF are limited, and comparisons are difficult due to methodologic differences including clinic- vs. population-based, cross-sectional vs. longitudinal, demographics (including age at diagnosis), rate standardization, diagnostic confidence (self-report, claims-based, medical record confirmation), diagnostic criteria and coding, inclusion/exclusion of specific ILD types (e.g., sarcoidosis or IPF), and follow-up time. The largest study by Olson et al. [23] included sarcoidosis – which has granulomatous inflammation as its mechanism of fibrosis, quite different from other causes of fibrosing ILD [32]. By using only claims data, all sarcoidosis cases with any type of lung involvement were included, as claims data could not identify only those with ILD. Our data show how claims data alone can influence results. Including all sarcoidosis cases with any type of lung involvement increased the age- and sex-standardized prevalence rate for ILD in our study by nearly two-fold to 444.1/100,000 persons, and for PPF by 5% to 85.0/100,000 persons (Table 2 and Online Resource 5). These differences help to explain the wide range of reported prevalence and incidence rates for ILD and PPF, with prevalence rates ranging from 6-118/100,000 persons and 3–70/100,000 persons, respectively [23, 31, 33–35]. Crude age-specific prevalence rates were also different than US rates post-9/11 ILD prevalence rates were 95.4/100,000 persons (age 50–59 years) and 630.9/100,000 persons (age 60–69 years) compared with the same ages in the US, 72/100,000 persons and 162/100,000 persons, respectively [36].

The majority of occupational/environmental ILD have long latency periods (often decades) between exposures and disease [37]. Therefore, it is not surprising that we found the average time between WTC-exposure and ILD diagnosis to be 13 years. With continued follow-up this average time will certainly increase.

We found older age on 9/11 predicted ILD. Also similar to other studies, smoking status was a risk factor for ILD, with ever-smokers having over 2-fold greater rate of developing ILD compared with never-smokers [38, 39]. The presence of GERD prior to ILD diagnosis increased the rate of ILD nearly 5-fold. The pathophysiological relationship between GERD and ILD remains controversial, but one possibility is that recurrent micro-aspiration leads to chronic inflammation and fibrosis [40, 41]. In a cohort without WTC-exposure, GERD was a significant contributor to ILD in those who had GERD prior to an ILD (non-IPF) diagnosis [42].

We found no published studies with lung function data prior to the development of ILD or PPF. We hypothesized that lung function prior to diagnosis would be predictive of the development of ILD. First post-WTC-exposure FVC was lower in those who developed ILD compared with the whole cohort. However, after adjusting for confounders, our analyses did not confirm lung function or change in lung function (regardless of when measured) as a significant risk factor for the development of ILD. This important finding, if confirmed in other cohorts, would indicate that lung function within the normal range, or even lung function decreases shortly after major respirable exposures, would not be a factor for identifying those at risk for the development of ILD. The absence of such an association would place an unexpected and significant limitation on the ability for clinicians to identify those who might benefit from closer surveillance and early anti-inflammatory and/or anti-fibrotic treatment after exposure or ILD diagnosis.

Many of the toxic components identified at the WTC-site have been associated with ILD in non-WTC settings [9–11]. Years later, WTC studies have demonstrated persistent inflammation [43, 44], a known prodrome for pathogenic fibrotic response [45]. Together with early case reports of ILD and PPF among WTC-exposed participants, our finding of an increased prevalence of ILD among FDNY-WTC-responders compared with a general population (age- and sex-standardized) is not unexpected. Yet, we were unable to identify a WTC-exposure-response gradient for the development of ILD. A prior study from a different WTC-cohort (WTC Health Registry) found a similar incidence rate (36.7/100,000 person-years), but with a WTC-exposure-response gradient [14]. The WTC Health Registry cohort includes not only rescue/recovery workers, but also those who lived and worked in the surrounding area south of Canal Street. Their study had further notable differences from ours: ILD was based on self-reports obtained from a survey without medical record confirmation, used a different definition of WTC-exposure intensity, and included different types of WTC-exposed individuals who were less likely to have intense dust cloud exposure. The inability to demonstrate a dose-response effect in our study may be due to these differences as well as the long latency period between exposure and disease, complexity of the host inflammatory response, low incidence rate for ILD in midlife populations, and other non-WTC-exposures that occur during firefighting. Individual pre-9/11 firefighting exposure data were not available; however, when we used service years (an imperfect proxy), we found no significant association with ILD or PPF.

It is difficult to directly compare survival rates between our study and prior studies due to differences in multiple factors including methodology, age at diagnosis, ethnicity, type of fibrotic lung disease, follow-up time, comorbidities, healthy worker effect, and treatment availability. The median survival of IPF has been reported to range from 2 to 5 years [46, 47]. The actual range of survival for any individual IPF patient can vary, with up to 25% of patients living beyond 10 years, especially when diagnosed earlier [48–52]. Following ILD diagnosis, we observed an average follow-up of 8.5 years, longer than generally reported.

A limitation of our study is that CTs were obtained only in those with clinical indications – symptoms, abnormal spirometry, or abnormal chest radiograph. This results in the inclusion of more ever-smokers. Complete incidence and prevalence rates require CT-screening of asymptomatic non-smokers, a limitation we share with other published studies. However, given the unique nature of our cohort with annual monitoring and free diagnosis/treatment, we believe few cases would have been missed. Compared with other studies, this may have led to higher incidence and prevalence rates as well as earlier diagnosis. Another limitation is that we may be missing post-diagnosis FVC measurements among participants who were too ill to produce reliable spirometry/PFTs or who died before a scheduled spirometry. This would likely result in overestimation of the average post-diagnosis lung function values and result in misclassification of some persons with PPF as non-PPF. Likewise, post-diagnosis follow-up may not be comparable to prior studies as our diagnosis date was defined as the first CT with ILD findings [15]. An additional limitation, similar to most published studies, is that CTs and PFTs were not done at a single facility, and CT interpretations were qualitative clinical readings rather than quantitative analyses. However, all were done at highly regarded facilities; the requirement for repeat confirmatory scans provided added confidence; and our process mimics real-world clinical practice. Furthermore, our definition for ILD excluded isolated interstitial lung abnormalities (i.e., isolated, unilateral, or ground glass opacities without bilateral reticular findings). Other limitations include incomplete data on underlying conditions predisposing ILD, such as connective tissue disorders, other than sarcoidosis, or family histories. Furthermore, though consistent with current guidelines, diagnosis did not rely on histopathology, which was only available for eight participants (7/8 from lung transplant specimens), and notably all confirmatory of the fibrosing ILD diagnosis by CT. Lastly, a lack of socioeconomic, racial, ethnic, and sex diversity may limit the generalizability of our findings.

Major strengths include the prospective longitudinal design and the requirement for ILD confirmation on repeat CT. The FDNY-WTC cohort is a single center cohort with pre-9/11 health data confirming that all ILD cases occurred post-9/11. The cohort has a high retention rate because of labor-management support, free monitoring and treatment, and financial compensation provided by the WTC Victims Fund [53]. Third, the ability to include over two decades of follow-up of middle-aged participants allowed adequate time for ILD development, diagnosis, and progression. Fourth, annual monitoring for symptoms and lung function changes, with a low threshold for CT and referral to in-house pulmonologists, allowed for the identification and confirmation of each case and their progression without reliance on imprecise ICD-10/CPT diagnosis/claims codes as proxies. Finally, because we are the only study with longitudinal lung function measurements obtained before and after 9/11, we were able to examine the effect of lung function, with and without the influence of 9/11 exposure, on the development of ILD. This allowed us to demonstrate that lung function in any capacity was not a significant risk factor.

### Interpretation

Our study summarizes characteristics of those with post-9/11 ILD in the FDNY-WTC cohort. Risk factors for the development of ILD were identical to non-WTC cohort studies – age, smoking status, and GERD. Although we did not observe a significant WTC-exposure-response gradient, we observed a two-fold greater prevalence of ILD, even after excluding sarcoidosis, compared with non-WTC studies. Half the ILD cases progressed to PPF, highlighting the unique nature of WTC exposure intensity. Lung function, pre- or post-exposure, was not a predictor of developing ILD or PPF. Five years after diagnosis, those who developed PPF had greater lung function decline than ILD without PPF. While healthy-worker effects cannot be discounted, longer survival than generally reported may also be due to this no-cost program removing any financial barriers to early diagnosis and treatment and possible differences in the pathophysiology of PPF vs. IPF. Our findings indicate that continued longitudinal follow-up is necessary to assess if additional ILD cases occur with or without PPF, if CTs alone are adequate to predict final outcomes, and if survival time increases with newer treatments.

### Electronic Supplementary Material

Below is the link to the electronic supplementary material.

Supplementary Material 1

## Data Availability

The data that support the findings of this study may be available upon request to the corresponding author, David J. Prezant.

## Abbreviations

COVID-19: Coronavirus Disease 2019
CT: Computerized tomography
DLCO: Diffusing capacity of the lungs for carbon monoxide
FDNY: Fire Department of the City of New York
FVC: Forced vital capacity
GERD: Gastroesophageal reflux disease
ILD: Interstitial lung disease
PFT: Pulmonary Function Tests
PPF: Progressive pulmonary fibrosis
TLC: Total lung capacity
WTC: World Trade Center
