# Supplementary material for: Interstitial Lung Disease and Progressive Pulmonary Fibrosis: a World Trade Center Cohort 20-Year Longitudinal Study
Source: Lung. 2024 May 7;202(3):257–67. doi: 10.1007/s00408-024-00697-z (PMC11142940; doi:10.1007/s00408-024-00697-z)
Supplement: Supplementary file 1 — Supplementary Material 1 [file 408_2024_697_MOESM1_ESM.docx]

**Article Title:**Interstitial Lung Disease and Progressive Pulmonary Fibrosis: A World Trade Center Cohort 20-year Longitudinal Study

**Journal:** Lung

**Authors:**Krystal L. Cleven, Rachel Zeig-Owens, Alexandra K. Mueller, Brandon Vaeth,Charles B. Hall, Jaeun Choi, David G. Goldfarb, David E. Schecter,Michael D. Weiden,Anna Nolan,Steve Salzman, Nadia Jaber, Hillel W. Cohen, David J. Prezant

**Corresponding Author:**David Prezant, MD; Department of Medicine, Montefiore Medical Center; Bureau of Health Services, Fire Department of the City of New York; Department of Epidemiology and Population Health, Albert Einstein College of Medicine; Email: [David.Prezant@fdny.nyc.gov](mailto:David.Prezant@fdny.nyc.gov)

Online Resource 1: Demographic and clinical characteristics of post-9/11 ILD participants by Honeycombing/Traction Bronchiectasis status

|  | Honeycombing or Traction Bronchiectasis | No Honeycombing or Traction Bronchiectasis |
| --- | --- | --- |
| Total | 38 (47.5%) | 42 (52.5%) |
| Male Sex | 37 (97.4%) | 42 (100%) |
| Race |  |  |
| White | 38 (100%) | 38 (90.4%) |
| Black | 0 | 2 (4.8%) |
| Hispanic | 0 | 2 (4.8%) |
| Other | 0 | 0 |
| Mean age on 9/11 (SD) | 54.1 (7.8) | 51.7 (8.5) |
| Mean age at ILD Diagnosis (SD) | 66.3 (8.8) | 65.1 (9.2) |
| Mean years after 9/11 until diagnosis (SD) | 12.4 (4.5) | 13.5 (4.1) |
| Smoker |  |  |
| Ever | 27 (71.1%) | 29 (69.1%) |
| Never | 11 (28.9%) | 13 (30.9%) |
| Employment Class |  |  |
| EMS | 3 (7.9%) | 1 (2.4%) |
| Firefighter | 35 (92.1%) | 41 (97.6%) |
| GERD | 29 (76.3%) | 33 (78.6%) |
| Arrival Time |  |  |
| Morning of 9/11 | 1 (2.6%) | 8 (19.1%) |
| Afternoon of 9/11 | 15 (39.5%) | 18 (42.9%) |
| 9/12 | 8 (21.0%) | 6 (14.2%) |
| 9/13-9/24 | 12 (31.6%) | 8 (19.0%) |
| After 9/24 | 2 (5.3%) | 1 (2.4%) |
| Unknown | 0 (0) | 1 (2.4%) |
| Duration at WTC site ≥ 3 months | 10 (26.3%) | 13 (31.0%) |
| Duration at WTC site < 3 months | 28 (73.7%) | 29 (69.0%) |
| Spirometry Measures |  |  |
| Pre-9/11 FVC % Pred mean (SD)^a^ | 99.1 (15.3) | 93.3 (9.7) |
| First post-9/11 FVC % Pred mean (SD) | 88.4 (18.3) | 88.8 (9.2) |
| Most Recent FVC % Pred mean (SD)^b^ | 77.7 (24.5) | 84.1 (14.6) |
| Lung Function |  |  |
| FVC Decline ≥ 5%^c^ | 36 (94.7%) | 42 (100%) |
| DLCO Decline ≥ 10%^d^ | 19 (59.4%) | 12 (37.5%) |
| Deceased | 11 (29.0%) | 8 (19.1%) |
| On home oxygen | 11 (29.0%) | 4 (9.5%) |
| Anti-fibrotic medication | 15 (39.5%) | 2 (4.8%) |
| Radiographic Features^e^ |  |  |
| Honeycombing | 19 (50.0%) | 0 (0%) |
| Traction Bronchiectasis | 32 (84.2%) | 0 (0%) |
| Reticular Infiltrates | 37 (97.4%) | 42 (100%) |
| Subpleural and Basilar Predominance | 34 (89.5%) | 31 (73.8%) |

^a^Data available for 14 in honeycombing/traction bronchiectasis group and 26 in no honeycombing/traction bronchiectasis group

^b^Spirometry measurement closest to the end of follow-up

^c^Data available for 38 honeycombing/traction bronchiectasis group and 41 in no honeycombing/traction bronchiectasis group

^d^Data available for 32 honeycombing/traction bronchiectasis group and 32 in no honeycombing/traction bronchiectasis group

^e^Some participants have overlapping features

ILD, interstitial lung disease; SD, standard deviation; EMS, Emergency Medical Services; GERD, gastroesophageal reflux disease; WTC, World Trade Center; FVC, forced vital capacity; FVC % Pred, forced vital capacity percent predicted; DLCO, diffusing capacity of the lungs for carbon monoxide

Online Resource 2: Demographic and clinical characteristics of those with ILD or sarcoidosis with ILD in the case definition

|  | ILD Status | | PPF Status Among ILD Cases | |
| --- | --- | --- | --- | --- |
|  | No ILD | All ILD | PPF | Without PPF |
| Total | 14,539 | 85 | 43 | 42 |
| Male Sex | 14,092 (96.9%) | 84 (98.8%) | 43 (100%) | 41 (97.6%) |
| Race |  |  |  |  |
| White | 12,668 (87.4%) | 80 (94.1%) | 42 (97.7%) | 38 (90.5%) |
| Black | 781 (5.4%) | 3 (3.5%) | 1 (2.3%) | 2 (4.8%) |
| Hispanic | 952 (6.6%) | 2 (2.4%) | 0 | 2 (4.8%) |
| Other | 90 (0.6%) | 0 | 0 | 0 |
| Mean age on 9/11 (SD) | 40 (9.5) | 52 (8.4) | 52 (7.4) | 52 (9.4) |
| Mean age at ILD Diagnosis (SD) |  | 65 (9.9) | 65 (9.4) | 65 (10.5) |
| Mean years after 9/11 until diagnosis (SD) |  | 12.7 (4.4) | 12.1 (4.3) | 13.4 (4.5) |
| Smoker |  |  |  |  |
| Ever | 5564 (38.5%) | 58 (68.3%) | 31 (72.1%) | 27 (64.3%) |
| Never | 8883 (61.5%) | 27 (31.8%) | 12 (27.9%) | 15 (35.7%) |
| Employment Class |  |  |  |  |
| EMS | 2013 (13.8%) | 5 (5.9%) | 3 (7.0%) | 2 (4.8%) |
| Firefighter | 12,526 (86.2%) | 80 (94.1%) | 40 (93.0%) | 40 (95.2%) |
| GERD | 6871 (47.3%) | 65 (76.5%) | 31 (72.1%) | 34 (81.0%) |
| Arrival Time at WTC site |  |  |  |  |
| Morning of 9/11 | 2249 (15.5%) | 12 (14.1%) | 3 (7.0%) | 9 (21.4%) |
| Afternoon of 9/11 | 6613 (45.5%) | 34 (40.0%) | 21 (48.8%) | 13 (31.0%) |
| 9/12 | 2631 (18.1%) | 15 (17.7%) | 8 (18.6%) | 7 (16.7%) |
| 9/13-9/24 | 2492 (17.1%) | 20 (23.5%) | 10 (23.3%) | 10 (23.8%) |
| After 9/24 | 441 (3.0%) | 3 (3.5%) | 1 (2.3%) | 2 (4.8%) |
| Unknown | 113 (0.8%) | 1 (1.2%) | 0 (0) | 1 (2.4%) |
| Duration at WTC site ≥ 3 months | 7114 (48.9%) | 27 (31.8%) | 13 (30.2%) | 14 (33.3%) |
| Duration at WTC site < 3 months | 7425 (51.1%) | 58 (68.2%) | 30 (69.8%) | 28 (66.7%) |
| Spirometry Measures |  |  |  |  |
| Pre-9/11 FVC % Pred mean (SD)^a^ | 98.4 (12.8) | 94.9 (12.6) | 95.7 (13.0) | 94.3 (12.5) |
| First post-9/11 FVC % Pred mean (SD) | 94.1 (12.5) | 89.1 (14.1) | 90.7 (16.1) | 87.5 (11.7) |
| Most Recent FVC % Pred mean (SD)^b^ | 91.9 (65.4) | 79.7 (20.0) | 76.9 (23.1) | 82.5 (16.1) |
| Mean Change in FVC |  |  |  |  |
| Post-9/11 (95% CI) | -30.8 mL/Year  (-31.8, -29.8) | -36.3 mL/Year  (-43.2, -29.4) | N/A^f^ | N/A^f^ |
| Post-ILD-diagnosis (95% CI) |  | -74.6 mL/Year (-88.9, -60.3) | N/A^f^ | N/A^f^ |
| Lung Function |  |  |  |  |
| FVC Decline ≥ 5%^c^ |  |  | 42 (97.7%) | 40 (95.2%) |
| DLCO Decline ≥ 10%^d^ |  |  | 20 (46.5%) | 14 (33.3%) |
| Vital Status |  |  |  |  |
| Alive | 13,456 (92.5%) | 66 (77.7%) | 30 (69.8%) | 36 (85.7%) |
| Deceased^e^ | 1040 (7.2%) | 19 (22.4%) | 13 (30.2%) | 6 (14.3%) |
| Respiratory related death | 99 (10.5%) | 6 (42.9%) | 4 (40.0%) | 2 (50%) |
| Other causes of death | 840 (89.5%) | 8 (57.1%) | 6 (60.0%) | 2 (50%) |
| Total person time (years) | 255885.5 | 1081.5 | 643.3 | 880.3 |
| Mean person time (years) | 17.6 | 12.7 | 15.0 | 21.0 |
| On home oxygen |  | 15 (17.7%) | 15 (34.9%) | 0 (0) |
| Anti-fibrotic medication |  | 17 (20.0%) | 17 (39.5%) | 0 (0) |

^a^Data available for 11,318 non-ILD, 23 PPF and 28 people in non-PPF

^b^Spirometry measurement closest to the end of follow-up

^c^Data available for 39 PPF and 40 non-PPF

^d^Data available for 33 PPF and 31 non-PPF

^e^Cause of death data available for 938 non-ILD, 9 PPF and 4 non-PPF

^f^Too few cases to calculate

ILD, interstitial lung disease; PPF, progressive pulmonary fibrosis; SD, standard deviation; EMS, Emergency Medical Services; GERD, gastroesophageal reflux disease; WTC, World Trade Center; FVC, forced vital capacity; FVC % Pred, forced vital capacity percent predicted; DLCO, diffusing capacity of the lungs for carbon monoxide

Online Resource 3: Demographic and clinical characteristics of those with ILD or sarcoidosis with lung involvement of any type in the case definition

|  | ILD Status | | PPF Status Among ILD Cases | |
| --- | --- | --- | --- | --- |
|  | No ILD | All ILD | PPF | Without PPF |
| Total | 14,445 | 179 | 43 | 136 |
| Male Sex | 13,999 (96.9%) | 177 (98.9%) | 43 (100%) | 134 (98.5%) |
| Race |  |  |  |  |
| White | 12,593 (87.4%) | 165 (92.8%) | 42 (97.7%) | 123 (90.4%) |
| Black | 777 (5.4%) | 7 (3.9%) | 1 (2.3%) | 6 (4.4%) |
| Hispanic | 948 (6.6%) | 6 (3.4%) | 0 | 6 (4.4%) |
| Other | 89 (0.6%) | 1 (0.6%) | 0 | 1 (0.7%) |
| Mean age on 9/11 (SD) | 40 (9.5) | 44 (10.8) | 52 (7.4) | 41 (10.4) |
| Mean age at ILD Diagnosis (SD) |  | 55 (13.1) | 64 (9.4) | 52 (12.7) |
| Mean years after 9/11 until diagnosis (SD) |  | 10.7 (5.2) | 11.9 (4.7) | 10.3 (5.4) |
| Smoker |  |  |  |  |
| Ever | 5549 (38.7%) | 73 (40.8%) | 31 (72.1%) | 94 (69.1%) |
| Never | 8804 (61.3%) | 106 (59.2%) | 12 (27.9%) | 42 (30.9%) |
| Employment Class |  |  |  |  |
| EMS | 1998 (13.8%) | 10 (5.6%) | 3 (7.0%) | 7 (5.2%) |
| Firefighter | 12,437 (86.1%) | 169 (94.4%) | 40 (93.0%) | 129 (94.9%) |
| GERD | 6811 (47.2%) | 125 (69.8%) | 31 (72.1%) | 94 (69.1%) |
| Arrival Time at WTC site |  |  |  |  |
| Morning of 9/11 | 2235 (15.5%) | 26 (14.5%) | 3 (7.0%) | 23 (16.9%) |
| Afternoon of 9/11 | 6568 (45.4%) | 79(44.1%) | 21 (48.8%) | 58 (42.7%) |
| 9/12 | 2609 (18.1%) | 37 (20.7%) | 8 (18.6%) | 29 (21.3%) |
| 9/13-9/24 | 2481 (17.2%) | 31 (17.3%) | 10 (23.3%) | 21 (15.4%) |
| After 9/24 | 441 (3.1%) | 3 (1.7%) | 1 (2.3%) | 2 (1.5%) |
| Unknown | 111 (0.8%) | 3 (1.7%) | 0 (0) | 3 (2.2%) |
| Duration at WTC site ≥ 3 months | 7055 (48.8%) | 86 (48.0%) | 13 (30.2%) | 73 (53.7%) |
| Duration at WTC site < 3 months | 7390 (51.2%) | 93 (52.0%) | 30 (69.8%) | 63 (46.3%) |
| Spirometry Measures |  |  |  |  |
| Pre-9/11 FVC % Pred mean (SD)^a^ | 98.4 (12.8) | 98.9 (12.9) | 95.7 (13.0) | 99.6 (12.8) |
| First post-9/11 FVC % Pred mean (SD) | 94.1 (12.5) | 91.9 (13.1) | 90.7 (16.1) | 92.3 (12.1) |
| Most Recent FVC % Pred mean (SD)^b^ | 91.9 (65.6) | 84.6 (17.1) | 76.9 (23.1) | 87.1 (14.0) |
| Mean Change in FVC |  |  |  |  |
| Post-9/11 (95% CI) | -30.8 mL/Year  (-31.8, -29.8) | -31.2 mL/Year  (-37.7, -24.8) | N/A^f^ | N/A^f^ |
| Post-ILD-diagnosis (95% CI) |  | -43.9 mL/Year (-53.6, -34.3) | N/A^f^ | N/A^f^ |
| Lung Function |  |  |  |  |
| FVC Decline ≥ 5%^c^ |  |  | 42 (97.7%) | 129 (95.6%) |
| DLCO Decline ≥ 10%^d^ |  |  | 20 (46.5%) | 35 (25.7%) |
| Vital Status |  |  |  |  |
| Alive | 13,364 (92.4%) | 158 (88.3%) | 30 (69.8%) | 128 (94.1%) |
| Deceased^e^ | 1038 (7.2%) | 21 (11.7%) | 13 (30.2%) | 8 (5.9%) |
| Respiratory related death | 99 (10.6%) | 6 (40.0%) | 4 (40.0%) | 2 (40.0%) |
| Other causes of death | 839 (89.4%) | 9 (60.0%) | 6 (60.0%) | 3 (60.0%) |
| Total person time (years) | 255054.2 | 1888.6 | 643.3 | 2853.0 |
| Mean person time (years) | 17.6 | 10.6 | 14.9 | 21.0 |
| On home oxygen |  | 16 (8.9%) | 15 (34.9%) | 1 (0.7%) |
| Anti-fibrotic medication |  | 17 (9.5%) | 17 (39.5%) | 0 (0) |

^a^Data available for 11,234 non-ILD, 23 PPF and 112 people in non-PPF

^b^Spirometry measurement closest to the end of follow-up

^c^Data available for 39 PPF and 40 non-PPF

^d^Data available for 33 PPF and 31 non-PPF

^e^Cause of death data available for 938 non-ILD, 9 PPF and 4 non-PPF

^f^Too few cases to calculate

ILD, interstitial lung disease; PPF, progressive pulmonary fibrosis; SD, standard deviation; EMS, Emergency Medical Services; GERD, gastroesophageal reflux disease; WTC, World Trade Center; FVC, forced vital capacity; FVC % Pred, forced vital capacity percent predicted; DLCO, diffusing capacity of the lungs for carbon monoxide

Online Resource 4: Post-9/11 ILD and PPF Prevalence and Incidence Rates, including those with ILD or sarcoidosis with ILD in the case definition

|  | ILD | | PPF | |
| --- | --- | --- | --- | --- |
| **Crude Age-Specific** | 2020 Prevalence Rates per 100,000 persons (95% CI)^a^ | Incidence Rates per 100,000 person-years (95% CI)^b^ | 2023 Prevalence Rates per 100,000 persons (95% CI)^c^ | Incidence Rates per 100,000 person-years (95% CI)^d^ |
| 40 to 49 years | 0 | 5.6  (2.3, 13.4) | 0 | 0 |
| 50 to 59 years | 132.0  (62.9, 277.1) | 25.2  (16.2, 39.0) | 20.6  (2.9, 146.4) | 4.2  (1.6, 11.1) |
| 60 to 69 years | 696.2  (489.0, 991.1) | 91.9  (64.6, 130.7) | 154.5  (77.2, 309.1) | 38.9  (24.8, 60.9) |
| 70 to 79 years | 2241.3  (1519.6, 3306.1) | 306.4  (208.5, 450.3) | 955.8  (584.2, 1563.7) | 116.9  (70.4, 193.9) |
| 80 to 89 years | 3750.1  (1927.6, 7294.9) | 296.3  (95.4, 920.4) | 1243.8  (514.9, 3004.4) | 102.4  (25.6, 409.9) |
|  |  |  |  |  |
| Age- and sex-standardized | 311.2  (245.0, 377.3) | 37.2  (30.3, 44.0) | 91.2  (63.9, 118.4) | 11.6  (7.3, 15.9) |

Rates were standardized to the US Census Bureau 2014 National Population Projections

^a^ILD prevalence was estimated for all alive participants at the end of case ascertainment (3/1/2020).

^b^ILD incidence was estimated from 9/11 until 3/1/2020. Person time accrual for the incidence rate began on 9/12/2001 and ended at the earliest date of ILD diagnosis, death, or 3/1/2020 (case ascertainment).

^c^PPF prevalence was estimated for all alive participants at the end of the study (3/31/2023) as one could have progressed after the end of case ascertainment.

^d^PPF incidence was estimated from 9/12/2001 to the earliest of date of PPF, death, or 3/31/2023.

ILD, interstitial lung disease; PPF, progressive pulmonary fibrosis; WTC, World Trade Center; FDNY, Fire Department of the City of New York

Online Resource 5: Post-9/11 ILD and PPF Prevalence and Incidence Rates, including those with ILD or sarcoidosis with lung involvement of any type in the case definition

|  | ILD | | PPF | |
| --- | --- | --- | --- | --- |
| **Crude Age-Specific** | 2020 Prevalence Rates per 100,000 persons (95% CI)^a^ | Incidence Rates per 100,000 person-years (95% CI)^b^ | 2023 Prevalence Rates per 100,000 persons (95% CI)^c^ | Incidence Rates per 100,000 person-years (95% CI)^d^ |
| 30 to 39 years | 0 | 53.9  (36.1, 80.4) | 0 | 0 |
| 40 to 49 years | 681.1  (422.7, 1097.3) | 58.2  (44.3, 76.3) | 0 | 0 |
| 50 to 59 years | 1258.8  (987.4, 1604.8) | 45.3  (32.7, 62.8) | 20.6  (2.9, 146.4) | 4.2  (1.6, 11.1) |
| 60 to 69 years | 1036.5  (775.2, 1385.9) | 103.7  (74.5, 144.5) | 154.5  (77.2, 309.1) | 38.9  (24.8, 60.9) |
| 70 to 79 years | 2506.5  (1733.9, 3623.2) | 330.0  (227.7, 478.2) | 955.8  (584.2, 1563.7) | 116.9  (70.4, 193.9) |
| 80 to 89 years | 3750.1  (1927.6, 7294.9) | 296.3  (95.4, 920.4) | 1243.8  (514.9, 3007.4) | 102.4  (25.6, 409.9) |
|  |  |  |  |  |
| Age- and sex-standardized | 507.2  (432.7, 581.7) | 69.6  (59.7, 79.5) | 85.0  (59.6, 110.3) | 11.7  (7.4, 16.0) |

Rates were standardized to the US Census Bureau 2014 National Population Projections

^a^ILD prevalence was estimated for all alive participants at the end of case ascertainment (3/1/2020).

^b^ILD incidence was estimated from 9/11 until 3/1/2020. Person time accrual for the incidence rate began on 9/12/2001 and ended at the earliest date of ILD diagnosis, death, or 3/1/2020 (case ascertainment).

^c^PPF prevalence was estimated for all alive participants at the end of the study (3/31/2023) as one could have progressed after the end of case ascertainment.

^d^PPF incidence was estimated from 9/12/2001 to the earliest of date of PPF, death, or 3/31/2023.

ILD, interstitial lung disease; PPF, progressive pulmonary fibrosis; WTC, World Trade Center; FDNY, Fire Department of the City of New York
